# Influence of arthritis and non-arthritis related factors on areal bone mineral density (BMD_a_) in women with longstanding inflammatory polyarthritis: a primary care based inception cohort

**DOI:** 10.1186/1471-2474-11-106

**Published:** 2010-05-28

**Authors:** Stephen R Pye, Tarnya Marshall, Karl Gaffney, Alan J Silman, Deborah PM Symmons, Terence W O'Neill

**Affiliations:** 1 Arthritis Research UK Epidemiology Unit, The University of Manchester, Manchester Academic Health Science Centre, Manchester, UK; 2 Norfolk and Norwich University Hospitals Trust, Norwich, UK

## Abstract

**Background:**

The aim of this analysis was to determine the relative influence of disease and non-disease factors on areal bone mineral density (BMD_a_) in a primary care based cohort of women with inflammatory polyarthritis.

**Methods:**

Women aged 16 years and over with recent onset inflammatory polyarthritis were recruited to the Norfolk Arthritis Register (NOAR) between 1990 and 1993. Subjects were examined at both baseline and follow up for the presence of tender, swollen and deformed joints. At the 10^th ^anniversary visit, a sub-sample of women were invited to complete a bone health questionnaire and attend for BMD_a _(Hologic, QDR 4000). Linear regression was used to examine the association between BMD_a _with both (i) arthritis-related factors assessed at baseline and the 10^th ^anniversary visit and (ii) standard risk factors for osteoporosis. Adjustments were made for age.

**Results:**

108 women, mean age 58.0 years were studied. Older age, decreasing weight and BMI at follow up were all associated with lower BMD_a _at both the spine and femoral neck. None of the lifestyle factors were linked. Indices of joint damage including 10^th ^anniversary deformed joint count and erosive joint count were the arthritis-related variables linked with a reduction in BMD_a _at the femoral neck. By contrast, disease activity as determined by the number of tender and or swollen joints assessed both at baseline and follow up was not linked with BMD_a _at either site.

**Conclusion:**

Cumulative disease damage was the strongest predictor of reduced femoral bone density. Other disease and lifestyle factors have only a modest influence.

## Background

Data from hospital-clinic based studies suggest that patients with rheumatoid arthritis (RA) have a reduction in areal bone mineral density (BMD_a_) at both the hip and spine compared to non-RA controls [1-4]. Such studies of patients with established disease will tend to exclude subjects with less marked disease who may be managed in primary care, either from the outset or discharged from specialist care early during the disease course. We studied a sample of women ten years prospectively, recruited following the onset of inflammatory arthritis presenting to primary care, to determine the relative influence of both arthritis and non-arthritis related factors on BMD_a _at the spine and femoral neck 10 years after disease onset.

## Methods

The subjects included in this analysis were recruited to the Norfolk Arthritis Register (NOAR) between 1990 & 1993, a primary care based inception cohort of adults with early inflammatory arthritis [5]. At baseline, subjects were examined by a trained research nurse for the presence of tender, swollen and deformed joints and had blood taken for c-reactive protein (CRP) and rheumatoid factor measurement. They also completed the Health Assessment Questionnaire (HAQ) [6]. Subjects are followed prospectively including assessments at 5, 7 and 10 years. Radiographs of the hands were performed at 5 years on all subjects who consented, and then again at 10 years if erosive change was present on the 5 year radiograph. Radiographs were assessed according to the method of Larsen [7] by two independent observers. Overall scores were compared between the observers and if the scores differed by more than 10%, the films were re-examined by both observers jointly. A third person adjudicated if there was disagreement. A Larsen score of two or more for an individual joint indicated the presence of erosive change. Subjects are asked additionally at these visits about use of steroids and disease modifying anti-rheumatic drugs (DMARD's). NOAR subjects continue to attend for their 10^th ^anniversary assessment. A subset (108) of those who were among the first to attend agreed to take part in a bone health survey which comprised an interviewer administered questionnaire and bone densitometry at the spine and femoral neck. The questionnaire was derived from a previously validated questionnaire on osteoporosis lifestyle risk factors [8] and included questions concerning:

(i) level of physical activity undertaken at work or home during three periods of adult life: 15-25 years, 25-50 years and 50 years and over (response set = light/moderate/heavy/very heavy)

(ii) time spent walking or on a bicycle out of doors each day (response set = none/< 30 minutes/30 minutes to 1 hour/> 1 hour)

(iii) if ever smoked, the number of cigarettes smoked a day

(iv) alcohol consumption in the previous year (response set = every day/5-6 days per week/3-4 days per week/1-2 days per week/less than once a week/not at all).

It also included questions about age at menarche and menopause and use of both the oral contraceptive pill and hormone replacement therapy. The revised questionnaire was not formally validated. Height and weight were measured using standard equipment and body mass index (BMI) calculated. Bone densitometry (Hologic, QDR 4000) was performed at the spine and femoral neck sites. All aspects of the study were in compliance with the Helsinki Declaration and approved by the Norfolk Research Ethics Committee.

### Analysis

Linear regression was used to determine the association between bone mineral density (BMD_a_) at the spine and hip and the various arthritis and non-arthritis related factors with BMD_a _as the dependent variable and adjustments made for age. We looked at the association between arthritis related factors assessed at baseline and BMD_a_, and arthritis related factors assessed at 10 years and BMD_a_. We looked also at cumulative disease activity by combining clinical data available from the preceding assessment periods (baseline, 5, 7 and 10 years) and categorising these into tertiles. For CRP we categorised the number of times individuals had an elevated CRP level (assessed at baseline, 5 and 10 years). The results are expressed as β coefficients and 95% confidence intervals (CI). Statistical analysis was performed using STATA version 9.2 (StataCorp, 2007).

## Results

### Subject characteristics

A total of 108 women (mean age 58.0 years, standard deviation [SD] = 13.2) were included in the analysis. Mean height was 1.6 m, weight 57.5 Kg and body mass index 21.6 Kg/m^2^. Mean age at menarche was 12.8 years. Of the 38 women who had reached natural menopause, the mean age at menopause was 48.0 years. Mean BMD_a _was 0.76 g/cm^2 ^(SD = 0.11) at the femoral neck and 0.96 g/cm^2 ^(SD = 0.14) at the lumbar spine respectively. Fifty three percent of subjects reported walking or cycling for 1/2 an hour or more daily, while 47%, 61% and 49% of subjects reported undertaking heavy or very heavy physical activity between the ages of 15-25, 25-50 and 50 years and over respectively. Fifty one percent of subjects reported ever having smoked, while 23% reported consuming alcohol on more than one day per week. Sixty percent reported taking the oral contraceptive pill in the past and 39% were currently taking hormone replacement therapy (HRT). The median (interquartile range) swollen and tender joint counts at baseline were 7 (3-15) and 9 (4-19) and at 10 years were 2 (0-6) and 2 (0-8) respectively. The median (interquartile range) deformed joint count at 10 years was 2 (0-7). Mean CRP level at baseline and 10 years was 14.1 and 12.3 mg/L respectively. 71 (66%) subjects satisfied the American College of Rheumatology (ACR) tree criteria at baseline and 86 (80%) at 10 years. Forty eight percent of subjects reported having ever taken DMARDs and 18% reported having taken steroids during follow up. Of those who had hand radiographs performed at the 10^th ^anniversary visit (n = 36) the median (interquartile range) number of erosive joints was 6 (3-14).

### Determinants of BMD_a_

As expected BMD_a _decreased with age (per year) at both the femoral neck (β coeff = -0.003; 95%CI -0.005, -0.002) and lumbar spine (β coeff = -0.004; 95%CI -0.006, -0.002). The influence of non-arthritis related factors on BMD_a _are presented in Table 1. Increasing weight (per 10 kg) was associated with an increase in BMD_a _at both the femoral neck (β coeff = 0.030;95%CI 0.004, 0.056) and lumbar spine (β coeff = 0.034;95%CI 0.002, 0.066). Similarly, BMI was associated with a significant increase in BMD_a _at both measurement sites. By contrast there was no association between physical activity, either lifetime or current, as determined by walking or cycling daily, with BMD_a _at either the femoral neck or spine. Similarly there was no association between BMD_a _and smoking or alcohol consumption. Increasing age at menarche (per year) was associated with a reduction in BMD_a _at both the femoral neck (β coeff = -0.017; 95%CI -0.032, -0.002) and lumbar spine (β coeff = -0.018; 95%CI -0.036, -0.001). Age at natural menopause, use of the oral contraceptive pill and HRT did not appear to be linked with BMD_a _at either site.

**Table 1:** Influence of anthropometric, hormonal and lifestyle factors on BMD_a _at the spine and femoral neck.

	Femoral Neck (g/cm^2^)	Lumbar Spine (g/cm^2^)
	β^1 ^coefficient	β^1 ^coefficient
Height (per 10 cm)	-0.014 (-0.047, 0.019)	0.001 (-0.040, 0.042)
Weight (per 10 kg)	0.030 (0.004, 0.056)*	0.034 (0.002, 0.066)*
BMI (kg/m^2^)	0.008 (0.002, 0.014)*	0.008 (0.001, 0.016)*
Menarche (years)	-0.017 (-0.032, -0.002)*	-0.018 (-0.036, -0.001)*
Age at natural menopause (years)	0.001 (-0.004, 0.005)	0.005 (-0.001, 0.010)
Oral contraceptive pill use: yes vs no	0.005 (-0.051, 0.060)	-0.002 (-0.069, 0.065)
Hormone replacement therapy: yes vs no	0.027 (-0.023, 0.077)	0.036 (-0.027, 0.100)
Walking or cycling: >= 1/2 hr vs < 1/2 hr	0.040 (-0.005, 0.085)	-0.005 (-0.059, 0.050)
Activity aged 15-25 yrs: heavy/very heavy vs light/moderate	0.036 (-0.008, 0.080)	0.031 (-0.021, 0.084)
Activity aged 25-50 yrs: heavy/very heavy vs light/moderate	0.021 (-0.026, 0.068)	0.001 (-0.057, 0.058)
Activity aged 50 yrs and over: heavy/very heavy vs light/moderate	-0.023 (-0.078, 0.033)	-0.055 (-0.122, 0.012)
Ever smoked: yes vs no	0.023 (-0.021, 0.066)	-0.008 (-0.059, 0.042)
Alcohol: >= 1 dy/wk vs < 1 dy/wk	0.032 (-0.019, 0.083)	-0.001 (-0.062, 0.061)

^1^adjusted for age.

*p < 0.05

The influence of arthritis-related factors assessed at both baseline and 10 years on BMD_a _are shown in Table 2. Disease activity expressed as active (swollen, tender or both) counts assessed either at baseline or at the 10^th ^anniversary visit showed no association with BMD_a_. Those with a CRP greater than 10 either at baseline or follow up had higher BMD_a _(at the 10^th ^anniversary) at both the spine and femoral neck though the confidence intervals embraced unity. Cumulative disease activity (swollen, tender joint counts) were not linked with BMD_a _(data not shown). For CRP, compared to those with CRP levels < 10 throughout, those who had elevated levels on one, or two or more occasions had higher BMD_a _at the femoral neck (p < 0.05). DMARD use and steroid therapy (both ever and also when expressed as duration of time on therapy), were not linked with BMD_a _at either site. Rheumatoid factor assessed either at baseline or 10 years was unrelated to BMD_a_. Disability as measured using HAQ was not linked with BMD_a_. Not all the subjects in this cohort satisfied ACR tree criteria for RA, either at baseline or by the 10^th ^anniversary. However stratification by 'RA' status also showed no association with BMD_a_. By contrast measures of structural damage were linked. Thus increased deformed joint count, as a measure of cumulative joint damage was linked with a reduction in BMD_a _at both sites. The effect was large, equivalent to approximately a 0.5 SD reduction for those above the lowest tertile of joint count and this was significant at the femoral neck. In the sub-sample of women with hand radiographs available those in both the mid and upper tertile of erosive joint count had lower BMD_a _at both the hip and spine than those in the lower tertile though the result was significant for those in the middle tertile only.

**Table 2:** Influence of disease related factors on BMD_a _at the spine and femoral neck.

	Baseline		10^th ^Anniversary	
		
	Femoral Neck (g/cm^2^) β^1 ^coefficient	Lumbar Spine (g/cm^2^) β^1 ^coefficient	Femoral Neck (g/cm^2^) β^1 ^coefficient	Lumbar Spine (g/cm^2^) β^1 ^coefficient
Swollen joint count:				
Lower	Referent	Referent	Referent	Referent
Mid	-0.043 (-0.096, 0.009)	0.003 (-0.061, 0.066)	-0.015 (-0.068, 0.039)	-0.061 (-0.123, 0.001)
Upper	-0.024 (-0.073, 0.025)	-0.012 (-0.071, 0.048)	-0.018 (-0.071, 0.035)	-0.020 (-0.081, 0.040)
Tender joint count:				
Lower	Referent	Referent	Referent	Referent
Mid	0.001 (-0.051, 0.052)	-0.020 (-0.081, 0.042)	-0.025 (-0.075, 0.026)	-0.015 (-0.075, 0.045)
Upper	-0.023 (-0.076, 0.029)	-0.027 (-0.090, 0.036)	0.004 (-0.051, 0.058)	-0.007 (-0.070, 0.057)
Both swollen & tender jt count:				
Lower	Referent	Referent	Referent	Referent
Mid	0.022 (-0.030, 0.074)	-0.007 (-0.070, 0.056)	-0.012 (-0.078, 0.055)	0.020 (-0.059, 0.098)
Upper	-0.009 (-0.059, 0.040)	0.002 (-0.058, 0.061)	-0.001 (-0.051, 0.048)	0.008 (-0.050, 0.066)
Deformed joint count:				
Lower	-	-	Referent	Referent
Mid			-0.047 (-0.098, 0.004)	-0.059 (-0.121, 0.003)
Upper			-0.067 (-0.117, -0.018)*	-0.046 (-0.106, 0.015)
Erosive joint count:				
Lower	-	-	Referent	Referent
Mid			-0.084 (-0.160, -0.008)*	-0.126 (-0.213, -0.039)*
Upper	-	-	-0.031 (-0.100, 0.039)	-0.014 (-0.093, 0.066)
CRP level:				
Normal <10 mg/L	Referent	Referent	Referent	Referent
Elevated ≥10 mg/L	0.044 (-0.003, 0.091)	0.041 (-0.017, 0.100)	0.031 (-0.013, 0.075)	0.030 (-0.026, 0.086)
HAQ score (0-3)	-0.004 (-0.034, 0.027)	-0.003 (-0.040, 0.034)	-0.003 (-0.031, 0.025)	-0.010 (-0.043, 0.024)
Steroid use (ever vs never)	-	-	0.006 (-0.055, 0.067)	0.008 (-0.065, 0.081)
DMARD use (ever vs never)	-	-	0.016 (-0.035, 0.068)	-0.016 (-0.079, 0.048)

^1^adjusted for age. *p < 0.05

## Discussion

In this inception cohort of women presenting to primary care with inflammatory polyarthritis there were several interesting findings relating to BMD_a _assessed after 10 years. Standard osteoporosis risk factors apart from age and body mass had relatively little impact. Of the disease factors considered those relating to activity and disability were not predictive of bone loss, whereas measures of joint damage were strongly predictive, especially at the femoral neck.

There are several limitations which need to be considered in interpreting the results. The subjects were deliberately selected to include all cases of inflammatory polyarthritis and not restrict inclusion to those with RA. Indeed we have shown that assigning criteria for RA is unstable in this setting during the first 5 years of disease [9]. As shown above however the results are stable independent of RA status. In this study we looked at BMD_a _in a subset of subjects attending their 10^th ^anniversary visit. It is possible that those who had BMD_a _performed may have differed from those who did not. However, those scanned did not differ in terms of joint count (swollen joint count mean = 3.9 vs 3.1; p = 0.11), HAQ score (mean = 1.02 vs 1.04; p = 0.82), or CRP level (mean = 12.3 mg/L vs 13.3; p = 0.59), from those who did not providing some reassurance against selection bias though we can not exclude this. Hand radiographs at the 10^th ^anniversary were only available on a proportion of the subjects. In order to have a 10^th ^anniversary radiograph, erosive change had to be present on the 5^th ^anniversary radiograph, so it is likely that these subjects had more severe disease. Indeed we found these subjects to be slightly older, have more joint involvement, higher CRP levels and HAQ score compared to those who did not have a 10^th ^anniversary hand radiograph. We did not have data on current smoking or OCP use at the time of bone density assessment or during the 10 year period of the study, so the influence of these could not be assessed. Finally the sample size was relatively small and the study had limited power to detect weak associations.

The lack of influence of disease activity on bone loss might be explained by measurement imprecision in relation to the joint counts. In an attempt to minimise this all of the assessments were undertaken by trained research nurses; annual comparative assessments were conducted to ensure standardisation of assessment. Four patients (with active joints) were invited to take part in the assessment process. Each nurse assessed each patient together with an independent observer. The results were compared, discrepancies discussed and repeat examinations performed as necessary to ensure agreement. Further any such imprecision should have applied equally to deformed joint counts which are perhaps harder to standardise, yet which showed a strong influence on BMD_a_.

Data from some though not all studies of patients with RA have suggested an association between disease activity assessed using erythrocyte sedimentation rate (ESR) or CRP and reduced bone mass [4,10,11]. In our long term follow up study, we observed a trend towards a positive association between CRP and bone mass, though the association did not attain statistical significance. This positive association has been reported previously though the mechanism is unclear [4].

In contrast to some though not all studies [2,10,12-15], we found no influence of corticosteroid use. We looked, however, only at ever use of steroids during the follow up period. There was insufficient information about dose of steroids used during the follow up period to allow assessment of the impact of current or cumulative dose in the study.

Previous studies have shown an inverse association with joint damage in RA as expressed by the Larsen score and BMD_a _by dual energy x-ray absorptiometry (DXA) [16-18]. In our study disease damage as determined both by the deformed joint count and erosive joint count at follow up was linked with increased bone loss. Such measures presumably represent a better marker of cumulative disease activity resulting over a long period in joint damage - though additional factors including immobility related bone loss may also be a factor. Many of the lifestyle factors investigated, such as smoking, exercise and hormonal use, had no significant influence on BMD_a _in this group. It is possible that associations with these variables were masked given the presence of longstanding arthritis. As expected based on studies of women without inflammatory arthritis, increasing age at menarche, weight and body mass index were linked with increased BMD_a _[19-21].

## Conclusions

In summary, in this group of women with longstanding inflammatory polyarthritis, cumulative disease damage was the strongest predictor of reduced bone density. Other disease and lifestyle factors had only a modest influence.

## Competing interests

The authors declare that they have no competing interests.

## Authors' contributions

SP performed the statistical analysis and drafted the manuscript. TM participated in the design and data collection of the study and contributed to the drafting of the manuscript. KG participated in the design and data collection of the study and contributed to the drafting of the manuscript. AJS conceived the study, participated in its design and coordination and helped to draft the manuscript. DPMS conceived the study, participated in its design and coordination and helped to draft the manuscript. TWO conceived the study, participated in its design and coordination and helped to draft the manuscript. All authors read and approved the final manuscript.

## Pre-publication history

The pre-publication history for this paper can be accessed here:

http://www.biomedcentral.com/1471-2474/11/106/prepub
